# Retraction Note: IMP3 accelerates the progression of prostate cancer through inhibiting PTEN expression in a SMURF1-dependent way

**DOI:** 10.1186/s13046-023-02599-z

**Published:** 2023-01-17

**Authors:** Xiang Zhang, Dawei Wang, Boke Liu, Xingwei Jin, Xianjin Wang, Junwei Pan, Weichao Tu, Yuan Shao

**Affiliations:** 1 Department of Urology, Ruijin Hospital, Shanghai Jiaotong University School of Medicine, No. 197, 2nd Ruijin Road, Shanghai, 200025 PR China; 2 Department of Urology, Ruijin Hospital North, Shanghai Jiaotong University School of Medicine, No. 999, Xiwang Road, Shanghai, 201801 China

**Retraction Note: J Exp Clin Cancer Res 39, 190 (2020)**

**https://doi.org/10.1186/s13046-020-01657-0**

The Editor in Chief and all authors have retracted this article. After publication, concerns were raised about the following issues with the images:

- Fig. 1C: the tumour-IHC:IMP3 panel appears very similar to the tumour-SAT1 panel in Fig 2N in a paper by different authors that was under submission at the same time [1].
- Fig. 2B: the LNCap IMP3 blot appears very similar to the SK-KEP-1 ACSL4 blot in Fig 2B in a previously-published paper by different authors [2].
- Fig. 2F contains multiple panels that seem to overlap with panels in Fig 1C a previously-published paper by different authors [3], Fig 1D in a previously-published paper by different authors [4], as well as Fig 5E, 6E and 7H in a paper by different authors that was under submission at the same time [1].
- Fig. 4A: the SMURF1 blot appears very similar to the N-Cahedrin blot in a paper by different authors that was under submission at the same time [5].
- Fig. 6F contains two tumours that appear similar to those in Figs 2M and 7H in in a previously-published paper by different authors [6].

The authors supplied the raw data, but in view of the multiple nature of the overlaps, the Editor in Chief has lost confidence in the integrity of the results of the article. All authors agree with this retraction.

## References

[1] Xiao Y, Li C, Wang H, Liu Y (2020). LINC00265 targets miR-382-5p to regulate SAT1, VAV3 and angiogenesis in osteosarcoma. Aging.

[2] Wang J, Wang Z, Yuan J, Wang J, Shen X (2020). The positive feedback between ACSL4 expression and O-GlcNAcylation contributes to the growth and survival of hepatocellular carcinoma. Aging.

[3] Sun L, Jiang C, Xu C, Xue H, Zhou H, Gu L, Liu Y, Xu Q (2017). Down-regulation of long non-coding RNA RP11-708H21.4 is associated with poor prognosis for colorectal cancer and promotes tumorigenesis through regulating AKT/mTOR pathway. Oncotarget **.

[4] Hu XM, Xiang JJ, Xiao BL, Huang YF, Xie JP (2019). Wogonoside promotes apoptosis in gastric cancer AGS and SGC-7901 cells through induction of mitochondrial dysfunction and endoplasmic reticulum stress. FEBS Open Bio.

[5] Ghosh Choudhury G, Chen Y, Zhang W, Shen L, Kadier A, Huang J, et al. Downregulation of Long Noncoding RNA LUCAT1 Suppresses the Migration and Invasion of Bladder Cancer by Targeting miR-181c-5p. Biomed Res Int. 2020:2314–6133. 10.1155/2020/4817608. 10.1155/2020/4817608 PMC7685804 33282949

[6] Yang J, Yu L, Yan J, Xiao Y, Li W, Xiao J, Yu X (2020). Circular RNA DGKB promotes the progression of neuroblastoma by targeting miR-873/GLI1 Axis. Front Oncol.

